# Radiotherapy and Cytokine Storm: Risk and Mechanism

**DOI:** 10.3389/fonc.2021.670464

**Published:** 2021-05-20

**Authors:** Chen Zhang, Zhenzhen Liang, Shumei Ma, Xiaodong Liu

**Affiliations:** ^1^ School of Public Health and Management, Wenzhou Medical University, Wenzhou, China; ^2^ NHC Key Laboratory of Radiobiology, School of Public Health, Jilin University, Changchun, China; ^3^ Key Laboratory of Watershed Science and Health of Zhejiang Province, Wenzhou Medical University, Wenzhou, China

**Keywords:** radiotherapy, cytokine storm, radiotherapy—adverse effects, immune activation, immune suppression, dose limitation

## Abstract

Radiotherapy (RT) shows advantages as one of the most important precise therapy strategies for cancer treatment, especially high-dose hypofractionated RT which is widely used in clinical applications due to the protection of local anatomical structure and relatively mild impairment. With the increase of single dose, ranging from 2~20 Gy, and the decrease of fractionation, the question that if there is any uniform standard of dose limits for different therapeutic regimens attracts more and more attention, and the potential adverse effects of higher dose radiation have not been elucidated. In this study, the immunological adverse responses induced by radiation, especially the cytokine storm and the underlying mechanisms such as DAMPs release, pro-inflammatory cytokine secretion and cGAS-STING pathway activation, will be elucidated, which contributes to achieving optimal hypofractionated RT regimen, improving the killing of cancer cells and avoiding the severe side effects.

## Cytokine Storm and Inducers

### Cytokine Storm

Cytokine storm, also named as cytokine release syndrome (1), hypercytokinemia (2), refers to an overactive immune response to external stimuli. Cytokine storm is first presented by Ferrara JL who hypothesized that inflammatory cytokines might act as mediators of acute graft versus host disease (GVHD) (3). The term is widely used in the research of severe acute infection and immunotherapy. Recently, studies have shown that not only viral infectious diseases, such as COVID-19 (4) and avian influenza (5), can induce cytokine storms, but many therapeutic interventions can also induce cytokine storms, such as Chimeric antigen receptor (CAR) T immunotherapy (1). It has been known that radiotherapy (RT) could elicit both immune activation and suppression responses (6, 7), however, if RT could induce cytokine storm or not, is still unclear.

### Different Inducers of Cytokine Storm

#### Graft Versus Host Disease

The understanding of the concept “cytokine storm” is relatively naïve in 1980s when it was first proposed (3, 8). Hill GR et al. hypothesized that most of the clinical manifestations of GVHD is due to the dysregulated production of cytokines by T cells and other inflammatory cells. Further study showed that the gastrointestinal (GI) tract is critical to the propagation of the cytokine storm because the GI tract increases the translocation of inflammatory stimuli such as endotoxin, which promotes further inflammation and additional GI tract damage, and the GVHD can be prevented by fortification of the GI mucosal barrier through novel “cytokine shields” such as IL-11 or keratinocyte growth factor successfully (9). Sato A et al. found that plasmin is activated during the early phase of acute graft-versus-host disease (aGVHD) and correlates with aGVHD severity. Plasmin inhibition could control the deadly cytokine storm in patients with aGVHD through impairing the infiltration of inflammatory cells or the release of membrane-associated proinflammatory cytokines including tumor necrosis factor-α and Fas-ligand directly. It could also relieve the cytokine storm *via* matrix metalloproteinases (MMPs) and alteration of monocyte chemoattractant protein-1 (MCP-1) signaling indirectly (10).

#### Severe Infection

Infectious disease is the second area of application of the concept “cytokine storm”. In the majority cases of Epstein-Barr virus (EBV)-associated hemophagocytic lymphohistiocytosis (EBV-HLH), clonally proliferating T-cells or NK-cells are involved. These cells produce massive cytokine followed by severe immune reactions for the host. To control the cytokine storm is important for the relieving of disease (11). Clinical data showed that cytokines such as interferon-gamma (IFNγ), interleukin-18 (IL-18), transforming growth factor β (TGF-β), interleukin-6 (IL-6) were highly elevated in the acute phase sera of severe acute respiratory syndrome (SARS) patients and their expression levels are related to the mortality (12). IFNγ could induce proliferation inhibition and enhancement of Fas-mediated apoptosis in alveolar epithelial cells and fibroblasts. These cells were able to secrete large quantities of T cell targeting chemokines and induced a Th1-type mediated cytokine storm in SARS patients (13). Clinical studies have detected cytokine storm in critical patients with COVID-19 and the cytokine storm is considered to be one of the major causes of ARDS and multiple-organ failure (4, 14). There are several mechanisms through which SARS-Cov-2 induces cytokine storm. Firstly, SARS-Cov-2 uses angiotensin converting enzyme II (ACE2) and transmembrane serine protease 2 (TMPRSS2) as cell entry receptors, ACE2 molecules on the cell surface are occupied by SARS-Cov-2. Then, angiotensin 2 (Ang II) increases in the serum due to a reduction of ACE2-mediated degradation. SARS-Cov2 activates NF-κB *via* pattern recognition receptors (PPRs), and the accumulated AngII induces inflammatory cytokines including TNFα and (s)IL-6R *via* disintegrin and metalloprotease 17 (ADAM17), followed by activation of the IL-6 amplifier (IL-6 AMP), which describes enhanced NF-κB activation machinery *via* the coactivation of NF-κB and transcription factor STAT3 (15). Secondly, Neutrophil extracellular traps (NETs), the extracellular NETs released by neutrophils can induce macrophages to secrete IL-1β which enhances NET formation in various diseases, the NET-IL-1β loop may contribute to cytokine release (16–19). Thirdly, IL-6 can work through classic cis signaling or trans signaling, in cis signaling IL-6 binds to membrane-bound IL-6 receptor (mIL-6R) in complex with gp130 and then activates acquired immune system (B and T cells) as well as the innate immune system (neutrophils, macrophage and NK cells); in trans signaling, high circulating concentration of IL-6 bind to the soluble (sIL-6R) also in complex with gp130 dimer on potentially all cell surfaces such as endothelial, this results in a systemic cytokine storm (20). Furthermore, cytokine storm may also occur in sepsis (14), dengue (21), influenza (22) etc.

#### Immunotherapy

CAR-T therapy is emerging as a promising new treatment for hematological and non-hematological malignancies (23), most remarkably in anti-CD19 CAR-T cells for B cell acute lymphoblastic leukemia (B-ALL) with up to a 90% complete remission rate (24). However, it may also induce rapid and durable clinical responses, such as cytokine storm. Features of CAR-T therapy-induced cytokine storm manifested as fever, hypotension and respiratory insufficiency associated with elevated serum cytokines such as IL-6. Cytokine storm usually occurs within days of T cell infusion at the peak of CAR T cell expansion and it is most frequent and more severe in patients with high tumor burden (25). In addition to CAR-T therapy, immunotherapy such as chimeric monoclonal anti-CD20 antibody rituximab (26), blinatumomab (27), nivolumab (28),brentuximab (29) etc. can also induce cytokine storm. Mechanically, cytokine storm is usually due to on-target effects induced by binding of the bispecific antibody or CAR T cell receptor to its antigen and subsequent activation of bystander immune cells and non-immune cells, such as endothelial cells. Activation of the bystander cells results in the massive release of a range of cytokines (1).

## RT and Immunological Effects

### RT Regimens

RT has been widely used for cancer treatment for more than a century (30). With the advance of clinical practice and the pursuit of a better prognosis, more and more types of RT have been developed. According to the fractionation way, the RT regimens can be divided into three types, conventional fractionated radiation, hypofractionated RT and hyperfractionated RT. Moreover, the RT regimens also contain 3-dimensional conformal radiation therapy (3D-CRT), intensity modulated radiotherapy (IMRT) and stereotactic body radiation therapy (SBRT), etc. Due to the higher local control rates, protection of local anatomical structure and relatively mild impairment, SBRT is widely used in the treatment of early-stage non-small cell lung cancer and localized pancreatic and prostate cancer in recent years (31). According to the most recent National Comprehensive cancer network (NCCN) guideline, RT is an option for patients with unresectable or inoperable HCC include external beam radiotherapy (EBRT) and SBRT. In patients with a limited number of liver or lung metastases, ablative RT to the metastatic site can be considered in highly selected cases or in the setting or clinical trial.

### The Killing Mechanisms Mediated by RT

The killing mechanism of ionizing radiation (IR) contains direct and indirect damage. IR can direct damage biomolecules, such as proteins and lipoids, particularly DNA, resulting in DNA double-strand breaks (DSBs) and other types of DNA damage (32). Indirect damage destroys biomolecules through free radicals, mainly by reactive oxygen species (ROS) (33, 34). The DNA damage response and repair (DRR) processes may determine tumor responses primarily (35). IR generates ROS through water radiolysis react with oxygen, high level of hydroxyl radicals induced by IR increases oxidative stress to destabilize cancer cells integrity and induces DNA damage, and subsequently results in cell death (33).

Cancer cells die in different ways after exposure to IR. Apoptosis, autophagic cell death, necrosis, and necroptosis are most common modalities that have been extensively studied and characterized. According to doses and cell types, IR might induce intrinsic apoptotic or extrinsic apoptotic pathway (36). Autophagy induced by IR is a double-edged sword, on one hand, it has a cytoprotective function allowing the cell to eliminate toxic species (37); on the other hand, it can serve as additional cell death pathway (38). Mitotic catastrophe is another modality of cell death induced by IR, resulting from premature induction of mitosis before completion of the S and G2 phase (39).

RT can be prescribed for curative or adjuvant therapy, depending on multiple factors especially the radiosensitivity of tumors. When certain tumors are treated at the early stage, for example, most lymphomas, carcinoma of the larynx, prostate or cervix, and some types of the central nervous system neoplasms, RT could be eutheraputic (34). With the gradual increase in high-dose hypofractionated RT applications, the indications of RT is expanding SBRT is an ablative radiation approach that has become an established standard of the treatment of a variety of malignancies, including intraabdominal malignancies such as primary and metastatic liver tumors and pancreatic tumors with excellent local control (40).

However, research regarding the impact of RT on the tumor immune microenvironment or systemic immune system is relatively fewer.

### Immune Activation Effects

**Figure 1:**
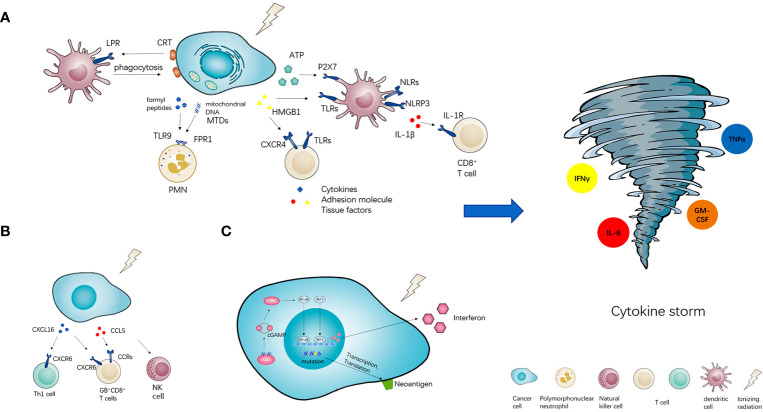
The potential of radiotherapy to induce cytokine storm. Radiotherapy (RT) has three or more ways of immune activation. **(A)** The first is damage- associated molecular patterns (DAMPs). RT could induce three types of DAMPs, namely exposed on the cell surface (CRT), released by cancer cells passively (HMGB1 and MTDs), secreted by cancer cells actively (ATP). DAMPs could interact with their pattern recognition receptors (PRRs) and activate downstream immune effects. **(B)** Meanwhile, radiotherapy could also stimulate the secretion of chemokines such as CXCL16 and CCL5, then recruit various pro-inflammatory immune cells into the tumor microenvironment. **(C)** Furthermore, radiotherapy could also arise the neoantigens presentation of neoantigens and active cGAS-STING signal pathway to increase the expression of interferon. All of the above immune activation effects could induce cytokine storm potentially.

As shown in **Figure 1A**, RT could induce damage- associated molecular patterns (DAMPs) (41). DAMPs can be divided into three classes, exposed on the cell surface including calreticulin (CRT), released by cancer cells passively (such as HMGB1 and mitochondrial DAMPs) (42), secreted by cancer cells actively (such as ATP). By interacting with their pattern recognition receptors (PRRs) respectively, immunogenic cell death (ICD) of cancer cells was induced (43). The most common PRRs include transmembrane proteins such as Toll-like receptors (TLRs) and C-type lectin receptors (CLRs), cytoplasmic proteins such as Retinoic acid-inducible gene (RIG)-I-like receptors and NOD-like receptors (NLRs) (41).

CRT is a soluble protein in the lumen of the endoplasmic reticulum (ER). In the ER, CRT has several functions, including chaperone activity and the regulation of Ca^2+^ homeostasis and signaling. CRT also assists in the proper assembly of major histocompatibility complex (MHC) class I molecules and the loading of antigen. Outside the ER, CRT regulates nuclear transport, cell proliferation and migration. A proportion of CRT on the plasma membrane of viable cells (ecto-CRT) serves various non-immunological functions. Ecto-CRT is an important signal that enables phagocytes to efficiently engulf dead cells. And the exposure of CRT on the surface of cancer cells also facilities the engulfment by dendritic cells, which leads to tumor antigen presentation and tumor-specific cytotoxic T lymphocyte (CTL) responses (43, 44). HMGB1, a member of the high mobility group (HMG) protein family, is a DNA-binding nuclear protein (45). It could bind multiple receptors including receptor for advanced glycation end-products (RAGE), TLRs (such as TLR2,4,7 and 9) etc. HMGB1 could stimulate different immune cells to produce a variety of inflammatory-related proteins, such as cytokines, adhesion molecules and tissue factors through the activation of several pathways (46, 47). Mitochondrial DAMPs (MTDs) include formyl peptides and mitochondrial DNA, they activate human polymorphonuclear neutrophils (PMNs) through formyl peptide receptor-1 and TLR9, respectively. Mechanically, MTDs promote PMN Ca^2+^ flux and phosphorylation of mitogen-activated protein (MAP) kinases, leading to PMN migration and degranulation *in vitro* and *in vivo*. Furthermore, circulating MTDs can elicit neutrophil-mediated organ injury (42). ATP, one of the most ancient and conserved DAMPs, exerts its phlogistic activity mainly through activation of the P2X7 receptor which is an ATP gated ion channel expressed by most immune cells (48). When released into tumor microenvironment, ATP acts on P2X7 purinergic receptors and triggers the NOD-like receptor family, pyrin domain containing-3 protein (NLRP3), allowing for the secretion of interleukin-1beta (IL-1β) then primes IFNγ-producing tumor antigen-specific CD8^+^ T cell in mice (49).

RT can also induce the expression of pro-inflammatory factors such as cytokines and chemokines which recruit immune cells to local sites of cancer (**Figure 1B**). Matsumura S et al. found that in mouse and human breast cancer cells IR markedly enhanced the secretion of CXCL16, followed by the recruitment of CXCR6^+^ Th1 and activation of CD8 effector T cells *in vitro* and *in vivo* (50). Tumor-infiltrating leucocytes (TILs) isolated from locally advanced hepatocellular carcinoma after Yttrium-90 (Y90)-radioembolization (RE) exhibited signs of local immune activation including higher expression of granzyme B (GB) and infiltration of CD8^+^ T cells, CD56^+^ NK cells and CD8^+^CD56^+^ NKT cells. Chemotactic pathways involving CCL5 and CXCL16 correlated with the recruitment of activated GB^+^CD8^+^ T cells to the Y90-RE-treated tumors (51).

RT is well suited for transforming poorly immunogenic tumors based on the tumor neoantigens. Radiation can increase existing tumor neoantigens through either radiation-induced transcription or increase antigen presentation, or RT induces neoantigens creation owing to DNA damage-induced mutations (52). 10 Gy RT can significantly increase the production of IFNγ through CD8^+^T cells, IFNγ inducible chemokines (CXCL9 and CXCL10) are increased with RT *in vitro* and *in vivo* (53). RT-induced micronuclei activate cytosolic nucleic acid sensor pathways, such as cyclic GMP-AMP synthase (cGAS)-stimulator of interferon genes (STING), and propagation of the resulting inflammatory signals remodels the immune contexture of the tumor microenvironment (**Figure 1C**) (52).

### Immune Suppression Effects

Studies have also revealed that RT could recruit suppressive immune cells to tumor microenvironment including myeloid derived suppressor cell (MDSC) (54), regulatory T cell (Treg) (55), tumor associated macrophage (TAM) (6), N2 neutrophils (56) etc. T cell activation could be repressed through these recruitments. IR can induce the secretion of granulocyte-macrophage colony-stimulating factor (GM-CSF) which promotes the migration of MDSCs to the circulatory system and to inflammatory tissue. MDSCs produce high levels of Arg1, which suppresses the activation and function of T cells through the degradation of arginine and expression reduction of the zeta chain of the CD3 complex, MDSCs may also limit the availability of cysteine and produce ROS that destroy T cell receptors, they can also trigger the PD-L1 pathway or IL-10 secretion (6). Similarly, the presence of Tregs may affect the efficiency of RT. RT significantly increased tumor-infiltrating Tregs (TIL-Treg), which had higher expression of CTLA-4, 4-1BB, and Helios compared with Tregs in non-irradiated tumors. TIL-Treg from irradiated tumors had equal or improved suppressive capacity compared with non-irradiated tumors. Tregs proliferate more robustly than other T-cell subsets in the TME and the increased Treg frequency is likely due to preferential proliferation of intratumoral Treg after radiation (57). RT can also upregulated CCL2 chemokine in tumor cells, leading to a CCR2-dependent accumulation of tumor necrosis factor alpha (TNFα)-producing monocytes and CCR2^+^ regulatory T cells (Treg) in a murine model of head and neck squamous cell carcinoma (55). Ovarian cancer cells and microenvironmental macrophages produce the chemokine CCL22 which mediates trafficking of Treg cells to the tumor (58). Radiation-induced 12-LOX overexpression in esophageal cancer cells (ESCC) upregulates CCL5 expression, thereby attracting THP-1-derived macrophages and promoting their polarization to the M2 subtype, consequently enhances cellular metastasis (59). Furthermore, radiation promotes secretion of TGF-β from tumor cells or increasing the expression of immune suppressive checkpoint molecules such as PD-L1 so that it has an immunosuppressive microenvironment (7).

### Immune Responses Based on Different Doses of Radiation

#### Low-Dose RT

There is no uniform standard for the definition of low-dose radiation (LDR). Radiation oncologists consider a single dose less than 1.0 Gy as a low one (60), United Nations Scientific Committee on the Effects of Atomic Radiation (UNSCEAR) defines it to be less than 100 mSv (61). Most studies show that LDR cannot trigger robust immune responses as high-dose radiation (62–64). LDR modulates a variety of immune response processes, and the regulatory effect of LDR on innate and adaptive immunity depends on the status of immune cells, the microenvironment and the interaction of immune cells ect (65). LDR suppressed release of mediator from mast cells activated by the antigen-antibody reaction *via* FcϵRI suppression (66). Neoadjuvant local low-dose gamma irradiation programs the differentiation of iNOS^+^ M1 macrophages that orchestrate CTL recruitment into and killing within solid tumors, the mechanisms include activation of endothelial, the expression of Th1 chemokines and the production suppression of angiogenic, immunosuppressive, tumor growth factors. All these effects eventually lead to T-cell-mediated tumor rejection and prolonged survival in human pancreatic carcinomas, immune refractor spontaneous and xenotransplant mouse tumor models (67). Yu N et al. found that X-ray irradiation (0.2 Gy) significantly increased CCR7- mediated DC migration and IL-12 production in dendritic cells (DCs), and the author identified ATM/nuclear factor kappaB (NF-κB) pathway as the central signaling pathway that mediated LDR-enhanced expression of IL-12 and CCR-7 (68). Our previous studies show that IL-12 level in macrophages increased after whole body irradiation (WBI) with 0.075 Gy x-rays in Kunming mice, which might contribute to a shift of the immune response in favor of Th1 differentiation (69, 70).

#### High-Dose RT

High-dose RT can induce necrosis and senescence which are considered more pro-inflammatory (71), for they are associated with release of damage-associated molecules (DAMPs) (72). SBRT can enhance the expression of intercellular and vascular adhesion molecules which associated with CTLs stimulation and binding effects by increasing IFNγ expression. Moreover, through releasing cytokines and adhesion molecules, SBRT can initiate the cellular immune response, enhance immune-cell extravasations and migration. It can also enhance antigen presentation by the pathway of OX40 stimulation (73). Ablative RT (20 Gy×1) dramatically increases T-cell priming in draining lymphoid tissues, leading to eradication of the primary tumor or distant metastasis in a CD8^+^ T cell-dependent pattern (63). Morisada M et al. found that compared with low-dose daily fractionated IR (2 Gy×10), high-dose hypofractioned IR (8 Gy×2) preserves or enhances anti-tumor immunity to control primary and distant tumors through accumulating and activating peripheral and tumor-infiltrating CD8^+^T-lymphocyte and reducing peripheral and tumor gMDSC accumulation (64). Similarly, they found that 8 Gy was superior to 2 Gy for induction of antigen-specific immune response and enhancing tumor cell susceptibility to T-lymphocyte killing in mouse oral cancer cells (74). Radiation in the ‘ablative’ doses range can not only effectively destroy tumor cells directly but also encourage these killed cells to function as an antitumor vaccine *in situ* (75).

### The Combinatorial Effect of RT and Immunotherapy

Recently, research on RT combined with immunotherapy has become more and more popular. The theoretical basis includes ‘in-situ vaccination’ and ‘abscopal effect’ of RT which are all connected to mechanisms involving the immune system, the combination of RT with immunotherapy potentially boost the killing of tumor (76, 77). The term ‘abscopal’ is used to describe an immune-medicated response to radiation by tumor cells located distant from the irradiated site (78, 79). Multiple preclinical studies have demonstrated that RT induces immunomodulatory effects in the local tumor microenvironment, different doses of hypofractionated radiotherapy have been shown to induce immunogenic cell death and in-situ vaccination in several tumor models (80), RT increases expression of tumor-associated antigens (TAAs), causes the release of cytokines, stimulates recruitment of dendritic cells and, most importantly, stimulates the proliferation and priming of cytotoxic CD8^+^ T cells in the tumor microenvironment (77), all these mechanism supporting a synergistic combination approach with immunotherapy to improve systemic control (81). Studies also found that RT could upregulate tumor PD-L1 expression, while the combination of RT and immune checkpoint inhibitors (ICIs) enhanced the anti-tumor effect of radiation consistent with the synergistic effect of both modalities (82–84). Furthermore, intratumoral injection of DCs (85), DC growth factor Flt3-ligand (86, 87), toll-like receptor 9 agonist C-G enriched synthetic oligodeoxynucleotide (CpG) (88) etc. all showed synergistic antitumor effects in preclinical research. Gratifyingly, clinical evidence such as case report and clinical trial showed that combination ICIs (cytotoxic T-lymphocyte associated protein 4 inhibitor, programmed cell death-1/programmed cell death ligand-1 blockers) with RT could improve progression-free survival of non-small cell lung cancer (NSCLC) patients (89, 90). No additional effect with concomitant regimens was found, furthermore, the combination of RT with ICIs seems better tolerated than radiotherapy combined with targeted or chemotherapy agents (77).

### Clinical Evidence for Cytokine Storm Induced by High-Dose RT

A case report from Memorial Sloan Kettering Cancer Center exhibits the capability of RT to elicit immune-related adverse events, i.e., cytokine storm/cytokine release syndrome. A 65-year-old man with untreated chronic lymphocytic leukemia (CLL) and recurrent, metastatic Merkel cell carcinoma undergoing anti-PD1 immunotherapy was referred for palliative RT (total dose of 24 Gy in three fractions given once weekly) to sites of progressing metastases. Within hours of each weekly dose of RT, he experienced fever, tachycardia, hypotension, rash, dyspnea, and rigors. Based on clinical suspicion for cytokine storm, blood cytokine measurements were performed 1 h after the second and third RT, TNF-α and IL-6 increased ten-fold higher. He experienced rapid regression of irradiated tumors, with development of new sites of metastases soon thereafter (91). The author inferred that RT produced tumor or tissue injury, released molecules that express DAMPs and caused activated macrophages to release the proinflammatory cytokines, then cause endothelial expression of adhesion molecules and leukocyte extravasation from the periphery at the site of RT. However, the underlying immune dysfunction caused by CLL, the ongoing immunotherapy with anti-PD1 therapy, MDSC depletion by RT and the advanced age of the patient may all have contributed to the observation of an atypical immune response to cancer therapy. Above all, this was the first report of cytokine storm after the receipt of RT and this case demonstrates the capability of RT to elicit immune-related adverse events.

As we mentioned earlier, RT could induce various immune activation responses (in-situ vaccination) including DAMPs release, pro-inflammatory cytokine secretion and cGAS-STING pathway activation etc. No evidence could rule out that RT would induce immune suppressive response. Under certain conditions such as immune system imbalance or combined with immunotherapy (91), an inflammation cascade can be involved and consequently lead to a cytokine storm.

## Prospect

With the increasing of RT dose, the adverse effect is what we concern. High-dose RT has an activating effect on the immune system. Moderate immune activation is beneficial for antitumor effect or therapy; however, overreaction might be harmful due to the possibility of superfluous pro-inflammatory cytokines secretion. Besides the efficacy, we must attach importance to the adverse immune response induced by radiation. Further research needs to confirm the existence of cytokine storm after RT and clarify the threshold of its occurrence to facilitate the dose limits setup in the clinic.

The mechanism of RT on the immune system is very complicated. RT could induce dual effects on immune response, which can be either activated or suppressive. However, the means to regulate different immune responses are not yet clear, for example dose and fractionation. Further research is needed to clarify the means in order to provide theoretical guidance for the rational use of its immune activation effect to achieve anti-tumor effects and to avoid its immune suppressive effect.

At present, RT combined with immunotherapy has entered the stage of clinical trials, but the following issues need to be solved before it is actually applied to the clinic: expanding the sample size of clinical trials to confirm safety and effectiveness; optimizing the complex immunological effects of RT and its compatibility with ICI interaction; treatment sequence and the issue of different doses and fractionation of RT, etc. Only by solving these problems can the two therapy be better integrated and their synergistic effects can be optimized.

## Data Availability Statement

The data used or analyzed during the current study are available from the corresponding author on reasonable request.

## Ethics Statement

This article does not contain any studies with human or animal subjects performed by any of the authors.

## Author Contributions

CZ was in charge of the writing of manuscript. ZL was in charge of editing the manuscript. SM was in charge of the revision of manuscript. XL conceived and designed this study, reviewed the manuscript, and was responsible for the overall content as a guarantor. All authors contributed to the article and approved the submitted version.

## Funding

Supported by National Natural Science Foundation of China (81972969, 81872558, 81773363 and 81673092).

## Conflict of Interest

The authors declare that the research was conducted in the absence of any commercial or financial relationships that could be construed as a potential conflict of interest.
